# Ultra-high dynamic range electro-optic sampling for detecting millimeter and sub-millimeter radiation

**DOI:** 10.1038/srep23107

**Published:** 2016-03-15

**Authors:** Akram Ibrahim, Denis Férachou, Gargi Sharma, Kanwarpal Singh, Marie Kirouac-Turmel, Tsuneyuki Ozaki

**Affiliations:** 1 INRS-EMT, Advanced Laser Light Source, Université du Québec, 1650 boul. Lionel- Boulet, Varennes J3X 1S2, Québec, Canada; 2 University of Massachusetts Lowell, 1 University Avenue, Lowell, 01854, Massachusetts, USA; 3 Harvard Medical School, Massachusetts General Hospital, 40 Blossom Street, Boston, 02114, Massachusetts, USA

## Abstract

Time-domain spectroscopy using coherent millimeter and sub-millimeter radiation (also known as terahertz radiation) is rapidly expanding its application, owing greatly to the remarkable advances in generating and detecting such radiation. However, many current techniques for coherent terahertz detection have limited dynamic range, thus making it difficult to perform some basic experiments that need to directly compare strong and weak terahertz signals. Here, we propose and demonstrate a novel technique based on cross-polarized spectral-domain interferometry to achieve ultra-high dynamic range electro-optic sampling measurement of coherent millimeter and sub-millimeter radiation. In our scheme, we exploit the birefringence in a single-mode polarization maintaining fiber in order to measure the phase change induced by the electric field of terahertz radiation in the detection crystal. With our new technique, we have achieved a dynamic range of 7 × 10^6^, which is 4 orders of magnitude higher than conventional electro-optic sampling techniques, while maintaining comparable signal-to-noise ratio. The present technique is foreseen to have great impact on experiments such as linear terahertz spectroscopy of optically thick materials (such as aqueous samples) and nonlinear terahertz spectroscopy, where the higher dynamic range is crucial for proper interpretation of experimentally obtained results.

Recent years have witnessed significant successful developments of techniques related to static and transient terahertz (THz) time-domain spectroscopy (THz-TDS) and imaging. The versatility of these techniques has been demonstrated in a wide range of applications in many disciplines, such as in spectroscopic imaging and tomography^1^,^2^, label-free genetic diagnostics and analysis^3^, biomolecular spectroscopy^4^, and biomedical applications such as T-ray biosensor^5^. Such remarkable progress has undoubtedly been accelerated by the incredible advances in both generating and detecting coherent THz radiation. For example, the recent surge in the availability of intense THz radiation sources is allowing new solutions to long-standing challenges, as well as opening up new avenues in both science and technology. For instance, intense THz sources have the potential to enable THz-TDS of aqueous samples, such as those related to biology and medicine^6^,^7^, with high signal-to-noise ratio (SNR)^8^. Due to the high absorption of THz radiation by water, such experiments have been difficult to perform up to now. However, the availability of THz sources with high flux is opening the possibility to perform THz-TDS of aqueous and other highly absorptive media *in transmission configuration*. Humidity also has significant influence in THz applications such as large-scale object imaging and remote sensing for security purposes^9^,^10^, and wireless communications^11^,^12^, in which the THz pulse may need to propagate long distances through the atmosphere. Intense THz sources are also enabling the observation of fascinating nonlinear phenomena in light-matter interaction process^13^,^14^,^15^,^16^,^17^,^18^,^19^,^20^. For example, THz nonlinear spectroscopy offers a unique characterization method of novel materials for future THz electronics, such as graphene^21^,^22^,^23^ and topological insulators^24^,^25^. In turn, such demand is pushing the limits of tabletop, intense, and ultrafast broadband THz emitting sources, resulting in peak THz electric fields of sub-MV/cm to several MV/cm^26^,^27^,^28^,^29^,^30^.

For such applications to be fully implemented, it is of crucial importance to have coherent THz detection techniques with both high dynamic range (DR) and high SNR^8^. High DR measurement is especially important in many of the above applications, where one needs to compare the waveform of intense THz pulses with relatively weak THz signals (such as the THz signal after experiencing strong absorption, or the nonlinear THz signal). Large DR is also required in THz-TDS measurements in order to avoid misinterpretation of experimentally obtained data^31^. However, electro-optic sampling (EOS)^32^,^33^,^34^, which is currently one of the most popular methods for THz detection, has achieved DR of only a few hundred. This limited DR of conventional EOS technique can be attributed to the mechanism by which the THz field is measured. In EOS, a linearly polarized femtosecond laser pulse co-propagates with a picosecond THz pulse in an electro-optic (EO) detection crystal^32^. The THz-electric-field-induced birefringence in the EO crystal (due to the Pockels effect) changes the polarization of the co-propagating optical probe pulse. The resulting ellipticity of the probe pulse is measured by using a detection system that consists of a quarter-wave plate, a polarizer and a pair of balanced photodiodes placed after the detection crystal. The change in phase, which is proportional to the THz electric field, is manifested as a modulation in the intensity of the two polarization components of the optical probe pulse. However, if the induced phase change in the probe pulse components is more than π/2, a reversal in the intensity modulation of the probe pulse will take place. This in turn results in ambiguities in measuring THz fields that induce phase differences of more than π/2^35^ in the conventional EOS configuration. This limitation is known as over-rotation^35^ and hence severely limits the DR of conventional EOS. In addition, over-rotation poses several other restrictions. For example, higher spectral resolution in THz-TDS requires thicker EO detection crystals^35^. However, a thicker crystal increases the possibility of over-rotation, as induced birefringence in the EO crystal is proportional to both the THz electric field and the thickness of the crystal. One could use thin (less than 50 μm) detection crystals, but the DR will still suffer from lower THz electric field modulation on the optical probe pulse.

Techniques such as air-biased-coherent-detection (ABCD)^36^,^37^ and spectral-domain interferometry (SDI)^35^,^38^ have been proposed and demonstrated as alternatives to the conventional EOS technique, to overcome such limited DR in THz-TDS. Improved DR of 4,400 has been reported with the ABCD technique, but this came with the cost of the need for expensive equipment and a much more complex setup, requiring the use of a high voltage source and a photomultiplier tube^36^. Meanwhile, with the SDI technique in THz detection, over-rotation and its consequent restrictions could be easily eliminated by unwrapping the phase induced by the intense THz signal when it is more than π/2, by using certain algorithms^35^. However, the standard SDI technique suffers from low SNR and limited temporal scan window, the latter being limited by the fixed optical path difference (OPD) between the two probe pulses^35^,^38^.

In the present work, we propose and demonstrate a new interferometric technique called cross-polarized spectral-domain interferometry (CP-SDI). Using this new technique, we have demonstrated a tremendous DR of 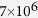 for the EOS detection of THz electric fields, while avoiding the limitation of over-rotation. Moreover, the limit in the temporal scan window suffered in the standard SDI technique in THz detection has been avoided, thus achieving SNR that are comparable to conventional EOS detection technique. This new technique makes use of the birefringence in polarization maintaining (PM) single-mode optical fiber in order to measure the phase change due to the THz electric field.

## Results

### Concept of the CP-SDI scheme

**Figure 1:**
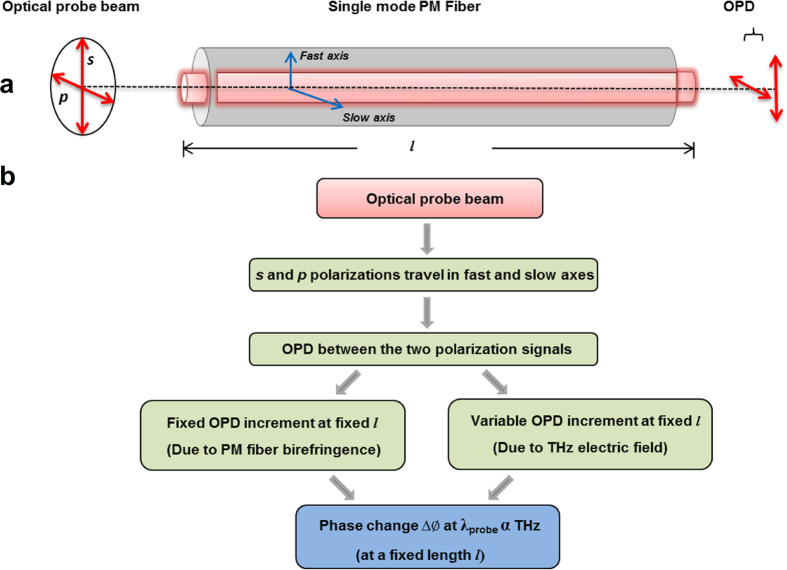
PM fiber introduces optical path difference. **(a)** Elliptically polarized optical probe beam after traversing the detection crystal is coupled into the single mode PM optical fiber where polarization components travel into fast and slow axes and at the output an OPD is introduced between the two signals **(b)** The evolution process of the optical fiber introducing a fixed OPD between the two polarization components. In the presence of THz signal, an additional OPD that is proportional to THz field strength is introduced between the two polarization states. The fixed OPD due to birefringence of the fiber will be systematic in the phase calculation while the variable OPD due to THz electric field induced birefringence in the ZnTe detection crystal is used to measure THz electric field pulse shape.

The major breakthrough of the present work is the design and demonstration of an effective scheme for improved THz electric field measurement with higher DR and SNR, which is crucial for various applications of THz technology, including spectroscopic measurements. In this new technique, we exploit the intrinsic phase delay between the fast and the slow axes of the birefringent fiber^39^ (see Fig. 1). The *s*- and *p*-polarization components of the probe pulse experience different refractive indices in the PM fiber, which results in the modification of the polarization state at its output, depending on the length of the fiber. In other words, the PM fiber creates two pulses; one propagating along the fast axis and the other propagating along the slow axis of the PM fiber, resulting in an OPD between the two. This is in addition to the OPD between the *s*- and *p*- components of the probe pulse that results from the birefringence introduced by the detection crystal, when the THz pulse and the probe pulse overlap in space and time. Then, we choose a common polarization direction that is neither of *s-* nor *p-*polarization (by using, for example a polarizer), to allow these two pulses to interfere. The OPD introduced by the THz electric field is measured from the interference signal of the two probe pulses as a change in the phase. The measured phase can be unwrapped by certain algorithms descried in ref. 35, leading to ultra-high DR for this technique.

The two orthogonal components of the probe pulse exiting the fiber cannot interfere, as they are cross polarized. In order to enable their interference, a polarizer is placed at the exit end of the PM fiber with its transmission axis at 45° with respect to the fast and slow axes. This allows a component of both *s*- and *p*-polarizations to pass through and to interfere in the spectrometer. At the CCD camera plane, the spectral components of the two pulses interfere, thus allowing one to measure the phase difference between them. Just as in the conventional EOS methods, the THz electric field induces birefringence in the ZnTe detection crystal, which is read out by the probe pulse when it overlaps with the THz pulse within the crystal in space and time. However, in the CP-SDI method, this birefringence is converted into a change in the OPD (or the phase) between the two probe pulses using a PM fiber and a polarizer. Using the interference signal between the spectral components of these two pulses; one can measure the THz electric field as a change in the phase difference introduced by the THz pulse.

The parameters of the PM optical fiber used in this study are summarized in Table 1.

### THz electric field measurement

**Figure 2:**
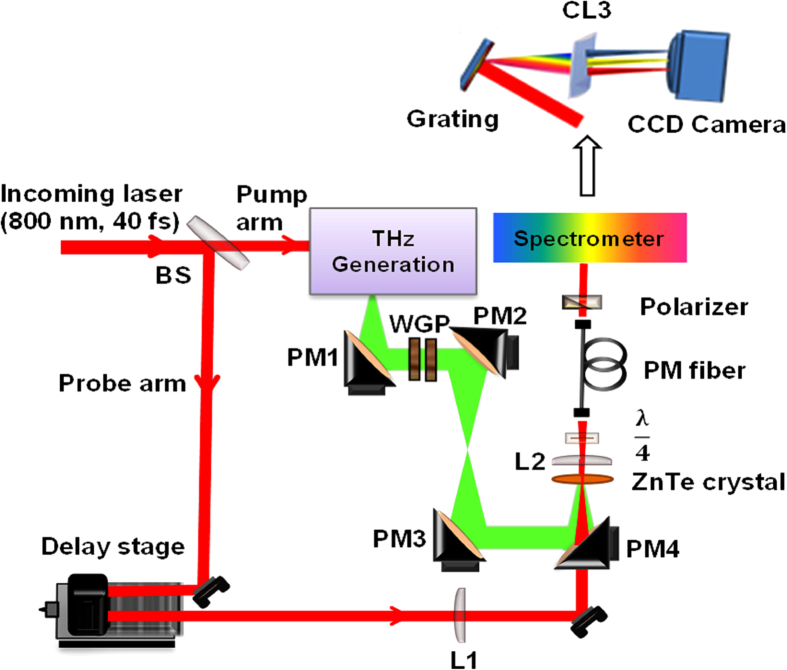
Experimental set up of the CP-SDI technique for THz detection. A beam splitter (BS) splits the beam into pump and probe arms for the generation and detection of THz, respectively. Spherical plano-convex lenses (L1, L2) focus and collimate the probe beam on and after the detection crystal respectively. Parabolic off-axis mirrors (PMs) guide the THz radiation to the detection crystal, where it spatially overlaps with the probe beam. Two wire grid polarizers (WGP) are used to control the electric field strength of THz radiation at the detection crystal. The spectrometer consists of a diffraction grating, a plano-convex cylindrical lens (CL3), and a two-dimensional (2D) CCD camera.

Here we experimentally compare THz electric field measurements obtained with the conventional EOS, the standard SDI, and the CP-SDI techniques. For the CP-SDI measurement, we have used the experimental set up shown in Fig. 2.

The resulting interference between the two component signals at the plane of the CCD camera can be expressed as^35^,^40^


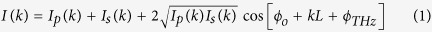


Here, *k* = 2π/λ is the wave number and λ is the central wavelength of the probe pulse, *I*_*p*_ and *I*_*s*_are the intensities of the *p*-polarization and *s*-polarization components of the probe pulse, respectively, *ϕ*_o_ is the phase constant, *L* is the optical path difference introduced between *s*- and *p*-polarization components by the PM fiber and *ϕ_THz_* is the phase introduced by the THz signal. The interference is recorded using a (2D) CCD camera and rescaled from wavelength space to wave-number (*k*) space and Fourier transformed to obtain the corresponding FFT spectrum. The instantaneous phase difference between the two signals is calculated using the relation


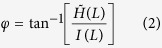


Here, 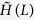 and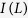 are the Hilbert transform and the interference signal intensity of equation (1) at an OPD of *L.* The change in the OPD over time can be traced by monitoring the phase change in equation (2). For a fixed length of the PM fiber, this phase changes only due to the birefringence induced by the THz electric field in the ZnTe detection crystal, which is proportional to the THz electric field strength. Hence the temporal shape of the THz electric field can be reconstructed by varying the delay time between the THz pulse and the optical probe pulse using a delay stage.

**Figure 3:**
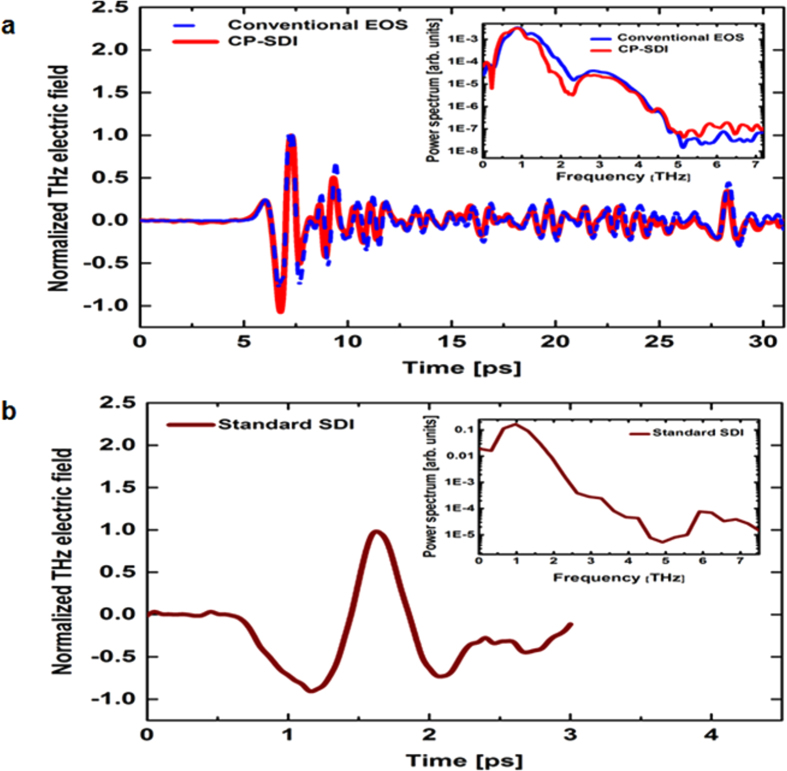
Terahertz electric field detection using the conventional EOS, the CP-SDI, and the Standard SDI techniques. A comparison between THz electric field traces obtained with **(a)** conventional EOS (blue dashed line), and the CP-SDI (red solid line), and **(b)** the standard SDI (wine solid line) techniques. The temporal scanning window in the case of the standard SDI is limited to 3 ps due to the thickness (300 μm) of the glass plate used in the technique. The inset in (**a**) shows the corresponding power spectra of the THz traces obtained with the conventional EOS and the CP-SDI techniques and in (**b**) shows the corresponding power spectrum of the THz trace obtained with the standard SDI technique.

We show in Fig. 3a the long temporal scans (>30 ps) for the traces of the THz electric field measured using the conventional EOS and the CP-SDI techniques. The trace obtained with the CP-SDI technique using an optical fiber length of 80 cm. At this length, the OPD between the two probe pulses will be ~400 μm (a temporal separation of ~1.3 ps) at a central wavelength λ_o_ = 790 nm when they exit the fiber. Worth to note that- in contrast to common interferometric methods- in SDI the two separated pulses in time interfere as two waves with different frequencies beat in time domain. Despite being temporally separated by more than the pulse duration, their spectral interference can be detected. The mode locked laser pulse is transformed into individual spectral components owing to the linear dispersion of the diffraction grating in the spectrometer. Several different directions will be taken by the different propagating spectral components, which in turn creates a frequency-dependent time delay^39^, therefore individual spectral components are spatially separated and hence are not mode locked anymore after the diffraction grating, consequently the two temporally separated pulses exiting the PM fiber can physically overlap on the CCD camera plane *i.e.* pulses interfere for much longer time scales.

For both THz traces in Fig. 3a, the main peak of the THz pulse is at 7.3 ps, while the second peak at 28.3 ps is the first reflection of THz pulse from the ZnTe detection crystal. For the sake of comparison we show in Fig. 3b a THz trace obtained with the standard SDI technique, where a 300 μm-thick glass plate was utilized to introduce a fixed OPD between the two probe pulses reflecting from its front and the back surfaces. One can see that the temporal scanning window is limited to only 3 ps owing to the fixed glass plate thickness in this version of SDI technique.

### Over-rotation limitation

**Figure 4:**
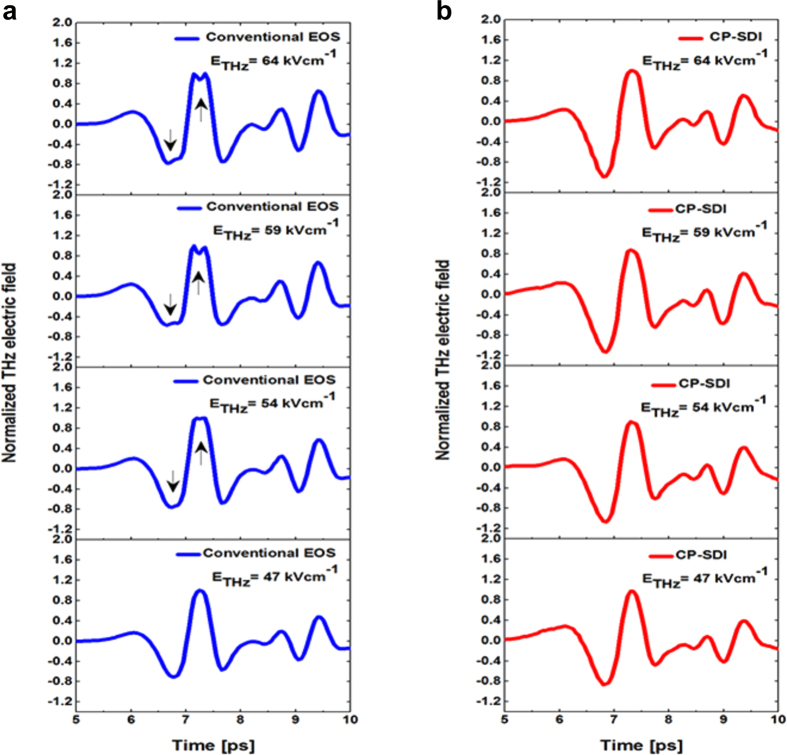
Short temporal scans of different THz electric fields demonstrating over-rotation effect. Short temporal scans of different THz electric fields (E_THz_) obtained with **(a)** the conventional EOS (blue line) and **(b)** the CP SDI (red line) techniques at various E_THz_ from 64 kVcm^−1^ to 47 kVcm^−1^. Over- rotation effect is evident in the conventional EOS (indicated by the arrows) at E_THz_ of 64 kVcm^−1^, 59 kVcm^−1^ and 54 kVcm^−1^. At lower E_THz_ 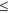 47 kVcm^−1^, the pulse obtained with the conventional EOS returns to its normal shape, while in the CP-SDI technique over-rotation effect has been avoided even at higher E_THz_ = 64 kVcm^−1^.

In order to compare the shape of the THz main pulse obtained with the conventional EOS and the CP-SDI techniques, short temporal scans (5 ps-long each) at different THz electric fields are shown in Fig. 4a,b. One can see that the THz pulse obtained at high THz electric fields (64 kVcm^−1^–54 kVcm^−1^) with the conventional EOS in Fig. 4a suffers from over-rotation in the parts indicated by arrows, which alter the shape of the pulse. At lower THz electric fields (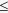 47 kVcm^−1^) the THz pulse obtained with the conventional EOS technique returns to its normal shape. Meanwhile when using the CP-SDI technique for the same THz electric fields, over-rotation is always avoided even at higher values of THz electric field, as shown in Fig. 4b.

### Signal-to-noise ratio

The inset in Fig. 3a shows the corresponding power spectra of the THz electric field obtained from the conventional EOS and the CP-SDI techniques. From these spectra, we find that the measured SNR is 48.97 dB for the CP-SDI technique compared to 52.17 dB for conventional EOS technique. Meanwhile, the inset in Fig. 3b shows the spectrum of THz pulse obtained with the standard SDI technique. The SNR measured to be 38.12 dB for the standard SDI technique compared to 48.97 dB for the CP-SDI technique.

We could also evaluate the SNR from the temporal shape of the THz pulse, by taking the ratio of the THz peak to the standard deviation of the baseline (signal that is before 1 ps) in the THz temporal trace. Following such approach, the SNR of the THz pulse measured with the CP-SDI technique (Fig. 3a) is ~279 compared to ~418 for the THz pulse obtained with the conventional EOS technique (Fig. 3a). At the same time, the SNR of THz pulse obtained with the standard SDI (Fig. 3b) is ~45.

### Dynamic range

For a Gaussian profiled spectrum, the maximum OPD that can be measured using the CP-SDI technique is given by^35^


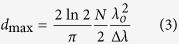


Applying the parameters of our system (central wavelength *λ*_o_ = 790 nm, bandwidth *∆λ* = 40 nm, and number of pixels of the CCD camera *N* = 640) in equation (3) yields a theoretical OPD range of 2.203 mm. Considering a change of 2π in the phase for one wavelength change in the OPD, the maximum overall phase change that we can measure with our CP-SDI system is 5,577π. In the conventional EOS technique, the maximum measurable phase change is π/2, which is approximately 11,000 times smaller than the maximum measurable phase change obtained with the CP-SDI technique. Moreover, the maximum achievable DR for our technique with our current setup is measured to be 7 × 10^6^, which is 4 orders of magnitude higher than the maximum achievable DR for our conventional EOS configuration of 6 × 10^2^. Meanwhile in the standard SDI the maximum achievable DR is 6 × 10^5^ when using the same spectrometer and laser source.

### Fiber length choice

**Figure 5:**
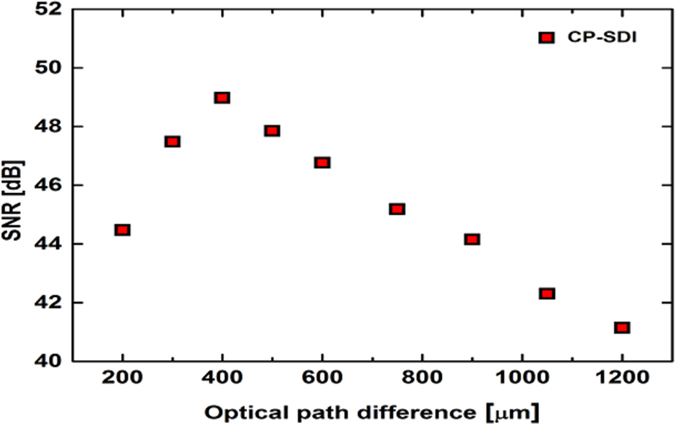
Change in SNR with length of the PM fiber. Dependence of the signal-to-noise ratio of the THz electric field measurements on the optical path difference between the two signals at the exit end of the optical fiber in the CP-SDI technique.

We further investigate the effect of fiber length on the SNR of our THz electric field measurement. For this purpose, PM fibers of different lengths, ranging from 40 to 240 cm (with corresponding OPD between the probe pulses at the exit end of the fiber of 200 to 1200 μm) were used. At each fiber length, a THz temporal trace was recorded for the same THz peak electric field (64 kVcm^−1^). Figure 5 shows the SNR measured using the CP-SDI THz detection technique for various OPD between the probe pulses. Our experimental findings show that the SNR increases as the OPD between the probe pulses decreases from 1200 μm to 400 μm. However, further decreasing the OPD from 400 μm to 200 μm reduces the SNR.

One could attribute this reduction in the SNR to the fact that working closer to the DC component of the interference signal (that is, closer to zero-OPD) will result in the signal subject to many low-frequency noises in the laboratory environment. On the other hand, the reduction in the SNR for OPD above 400 μm could be attributed to the reduced contrast of the interference fringes when working far away from zero-OPD where the sensitivity of the SDI technique is minimal. (See Supplementary Note 1 and Supplementary Figure S1 online).

## Discussion

The conventional EOS technique suffers from over-rotation, which was observed in our experiment design as well. However, by using our newly designed CP-SDI, we could circumvent this limitation while maintaining comparable SNR. By eliminating over-rotation, we could achieve a dynamic range of 7 × 10^6^. On the other hand, the standard SDI technique in THz electric field measurement presented in our earlier work^35^ achieved inferior SNR and the DR compared to SNR and DR of the new CP-SDI technique. In the standard SDI technique one of the major sources of phase noise is angular vibration in the glass plate owing to the angular variation between its two surfaces^35^. Furthermore the OPD between the two pulses reflecting from the glass plate surfaces was relatively large (900 μm) which results in working relatively far from zero-OPD which in turn cause a loss in interference signal of the two pulses and consequently lowers the SNR and the DR. However in the new CP-SDI technique, introducing the PM fiber increases the SNR in THz measurement primarily because of working closer to zero optical path difference where sensitivity of the technique is maximal. Moreover, by using the PM fiber, the noises associated with glass plate vibrations which change the optical path difference between two interfering signals, are eliminated. A change in optical path difference because of glass plate vibration in the standard SDI technique will result in an additional change in phase, on top of the phase change introduced by the THz electric field, and appears as noise. This noise is eliminated in our new CP-SDI technique, since both interfering signals are travelling through the same fiber and any change in OPD is common to both signals. The PM fiber also enables higher dynamic range since the interfering signals are generated after the detection crystal where as in the standard SDI technique, the interfering signals are generated before the detection crystal, limiting the scan length to the temporal thickness of the glass plate. Consequently the CP-SDI technique has achieved higher SNR and dynamic rage compared to the standard SDI technique.

Furthermore, the new CP-SDI technique in THz measurement eliminates the limit in the temporal scan length- crucially required for any spectroscopic application- which was a drawback of the standard SDI technique^35^ in THz detection due to limit imposed by the thickness of the glass plate, however in the new technique the PM fiber imposes no limit in the scan length. Indeed the PM fiber plays a critical role in adjusting a suitable working point on the OPD where the detection sensitivity is high and at the same time the noises are minimal. Alternatively this effect could be also achieved by using a birefringent crystal with appropriate dimensions; however that comes with the expenses of growing such crystal, whereas PM fibers are commercially available at affordable prices and can be manipulated easily in small size set ups.

In order to evaluate the capability of our technique to measure different THz peak electric fields with reasonable SNR, the THz peak electric field was varied from ~4 kVcm^−1^ to ~64 kVcm^−1^ by using two wire-grid polarizers. The amplitude THz electric field (E_THz_) is measured with conventional EOS technique using the modulation formula in equation (4)^26^,^27^.


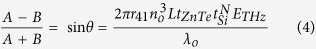


Here *A* and *B* are the signals from the balanced photodetectors in EO detection configuration, *θ* is the phase shift induced by the THz peak electric field, 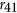 is the electro-optic coefficient of the ZnTe detection crystal, *n*_o_ is the refractive index of ZnTe, *L* is the ZnTe thickness, Fresnel transmission coefficients *t*_ZnTe_ for ZnTe and *t*_Si_ for the silicon wafer, *N* is the number of silicon wafers, and 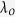 is the central wavelength.

At THz peak electric field of 64 kVcm^−1^ we could measure SNR of 48.97 dB for the THz electric field measurement obtained with CP-SDI technique compared to 52.17 dB obtained with conventional EOS technique. Meanwhile at THz field of 4 kVcm^−1^ we could measure SNR of 28.45 dB for the CP-SDI technique compared to 34.77 dB for the conventional EOS technique. These experimental findings are indicative of the potentials of the new technique in measuring higher THz electric fields as well as lower fields. Further enhancement in SNR of the new CP-SDI technique could be achieved if self-referencing is implemented in the setup, which has been proven to enhance the SNR considerably by reducing the noise due to vibration and laser fluctuations in the set up^38^.

In summary, we propose and demonstrate a new EOS based technique using PM optical fiber and SDI. The new technique has a dynamic range that is 4 orders of magnitude higher than the conventional EOS technique, while maintaining comparable SNR. The technique is promising and has the potential to significantly advance new areas of THz science and technology, such as nonlinear THz spectroscopy, thus opening new avenues in THz-TDS and imaging techniques that require large dynamic range.

## Methods

### CP-SDI scheme

Here we use the experimental set up illustrated in Fig. 2 where a femtosecond 800 nm laser beam is split by a beam splitter (BS) into two arms: the pump arm and the probe arm. The pump arm is used to generate the THz pulse. The probe beam is focused by a spherical plano-convex lens (L1), and then propagates through a hole in a parabolic off-axis mirror (PM4) and onto a 1-mm-thick <110>ZnTe detection crystal, where it overlaps with the focused THz pulse. Another spherical plano-convex lens (L2) is used to collimate the optical probe beam after the ZnTe detection crystal. A quarter-wave plate is used before a single-mode PM optical fiber (Thorlabs, PM 780-HP) to convert the linear polarization state of the probe pulse to circular polarization without the presence of the THz pulse. Then the probe pulse is coupled into the PM fiber, with its polarization components (*s*- and *p*-) set along the two orthogonal birefringent axes of the PM fiber depending on the birefringence introduced in the ZnTe crystal by the THz electric field. The wire grid polarizers are used to vary THz peak electric field. The custom-made spectrometer consists of a diffraction grating (1200 grooves/mm), a plano-convex cylindrical lens CL3 (*f *= 150 mm), and a two-dimensional (2D) CCD camera (Dalsa Inc., CR-GM00-H6400, 480 × 640 pixels). We measure the phase change between the spectral components of the two polarization signals exiting the single mode PM fiber at a fixed length, which is proportional to the THz electric field strength only. By varying the delay time between the THz pulse and the optical probe pulse using a delay stage the temporal shape of the THz electric field can be plotted. Worth to mention that the experimental data extraction is straightforward and carried out in real time by a LabVIEW code that is developed in our group, moreover the experiment does not require any longer time than that taken by the conventional EOS technique.

### Standard SDI scheme

Here we use the experimental setup described in our earlier work^35^ with a different CCD camera in the spectrometer (Dalsa Inc., CR-GM00-H6400, 480 × 640 pixels).

### THz generation and peak electric field calculation

THz generation throughout this work was achieved via the tilted-pulse-front technique in a LiNbO_3_ crystal^41^,^42^ pumped with 0.96 mJ pulses from Ti:Sapphire laser (repetition rate of 2.5 kHz, wavelength of 800 nm, pulse width of 40 fs, and pulse energy up to 4 mJ). Single cycle THz pulses with energies up to 0.3 μJ are generated with bandwidth extending from 0.1 to 3 THz. In order to evaluate the THz peak electric field, an electro-optical signal ∆*I* / *I* was measured with balanced photodiodes and calibrated to the THz electric field amplitude *E*_THz_ using the equation 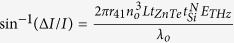 (ref. 26). Here, *n*_*o*_ *=* 2.87 (ref. 43) is the refractive index of ZnTe crystal, 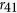 = 4.04 pmV^−1^(ref. 44) is the electro-optical coefficient of ZnTe, *t*_ZnTe_= 2/(*n*_THz_+1) = 0.479 is Fresnel transmission coefficients of ZnTe crystal where *n*_THz_ = 3.17 (ref. 45) is the refractive index of ZnTe crystal at 1 THz, *t*_Si_ = 0.7 (ref. 26) is Fresnel transmission coefficients of one silicon wafer, *L* (=1 mm) is the thickness of ZnTe crystal, *N* (=7) is the number of high-resistivity silicon attenuators used to reduce the THz field amplitude before ZnTe detection crystal, and λ_o_ (=790 nm) is the central wavelength.

## Additional Information

**How to cite this article**: Ibrahim, A. *et al*. Ultra-high dynamic range electro-optic sampling for detecting millimeter and sub-millimeter radiation. *Sci. Rep.* **6**, 23107; doi: 10.1038/srep23107 (2016).

## Supplementary Material

Supplementary Information

## Figures and Tables

**Table 1:** Parameters of the PM fiber used in the experiment.

Parameter	Value
Numerical aperture	0.12
Core diameter	4.5 μm
Attenuation	≤4 dB/km @ 850 nm
Operating wavelength	770–1100 nm
Second mode cut-off	710 ± 60 nm
Mode field diameter (1/e2fit – near field)	5.3 ± 1.0 μm @ 850 nm
Beat length	2.4 mm @ 850 nm
Birefringence	3.5 × 10^–4^

## References

[b1] Watanabe Y. . Component spatial pattern analysis of chemicals using terahertz spectroscopic imaging. Appl. Phys. Lett. 83, 800–802 (2003).

[b2] Wang S., Ferguson B., Abbott D. & Zhang X. C. T-ray imaging and tomography. J. Biol. Phys. 29, 247–256 (2003). 23345841 10.1023/A:1024457212578 PMC3456432

[b3] Nagel M. . Integrated THz technology for label-free genetic diagnostics. Appl. Phys. Lett. 80, 154–156 (2002).

[b4] Markelz A., Whitmire S., Hillebrecht J. & Birge R. THz time domain spectroscopy of biomolecular conformational modes. Phys. Med. Biol. 47, 3797–3805 (2002). 12452570 10.1088/0031-9155/47/21/318

[b5] Mickan S. P. . Label-free bioaffinity detection using terahertz technology. Phys. Med. Biol. 47, 3789–3795 (2002). 12452569 10.1088/0031-9155/47/21/317

[b6] Zhang X.-C. . Terahertz wave imaging: horizons and hurdles. Phys. Med. Biol. 47, 3667–3677 (2002). 12452553 10.1088/0031-9155/47/21/301

[b7] Pickwell E. & Wallace V. P. Biomedical applications of terahertz technology. J. Phys. D: Appl. Phys. 39, R301–R310 (2006).

[b8] Zhao G., Schouten R. N., Van der Valk N., Wenckebach W. T. & Planken P. C. M. A terahertz system using semi-large emitters: noise and performance characteristics. Phys. Med. Biol. 47, 3699–3704 (2002). 12452556 10.1088/0031-9155/47/21/304

[b9] Tonouchi M. Cutting-edge terahertz technology. Nature Photon. 1, 97–105 (2007).

[b10] Liu H.-B., Zhong H., Karpowicz N., Chen Y. & Zhang X.-C. Terahertz spectroscopy and imaging for defense and security applications. Proc. IEEE 95, 1514–1527 (2007).

[b11] Koenig S. . Wireless sub-THz communication system with high data rate. Nature Photon. 7, 977- 981 (2013).

[b12] Huang K.-C. & Wang Z. Terahertz terabit wireless communication. IEEE Microw. Mag. 12, 108–116 (2011).

[b13] Vicario C. . Off-resonance magnetization dynamics phase-locked to an intense phase-stable terahertz transient. Nature Photon. 7, 720–723 (2013).

[b14] Tanaka K., Hirori H. & Nagai M. THz nonlinear spectroscopy of solids. IEEE Trans. THz Sci. Technol. 1, 301–312 (2011).

[b15] Kubacka T. . Large-amplitude spin dynamics driven by a THz pulse in resonance with an electromagnon. Science 343, 1333–1336 (2014). 24603154 10.1126/science.1242862

[b16] Hwang H. Y. . A review of non-linear terahertz spectroscopy with ultrashort tabletop-laser pulses. J. Mod. Opt. 62, 1447–1479 (2015).

[b17] Kampfrath T. . Coherent terahertz control of antiferromagnetic spin waves. Nature Photon. 5, 31–34 (2011).

[b18] Razzari L. . Nonlinear ultrafast modulation of the optical absorption of intense few-cycle terahertz pulses in n-doped semiconductors. Phys. Rev. B 79, 193204 (2009).

[b19] Su F. H. . Terahertz pulse induced intervalley scattering in photoexcited GaAs. Opt. Express 17, 9620–9629 (2009). 19506611 10.1364/oe.17.009620

[b20] Liu M. . Terahertz-field-induced insulator-to-metal transition in vanadium dioxide metamaterial. Nature 487, 345–348 (2012). 22801506 10.1038/nature11231

[b21] Hafez H. A. . Nonlinear transmission of an intense terahertz field through monolayer graphene. AIP Adv. 4, 117118 (2014).

[b22] Hafez H. A. . Nonlinear terahertz field-induced carrier dynamics in photoexcited epitaxial monolayer graphene. Phys. Rev. B. 91, 035422 (2015).

[b23] Vicarelli L. . Graphene field-effect transistors as room-temperature terahertz detectors. Nat. Mater. 11, 865–871(2012). 22961203 10.1038/nmat3417

[b24] Zhang X., Wang J. & Zhang S.-C. Topological insulators for high-performance terahertz to infrared applications. Phys. Rev. B. 82, 245107 (2010).

[b25] Wu L. . A sudden collapse in the transport lifetime across the topological phase transition in (Bi_1−X_In_X_)_2_Se_3_. Nature Phys. 9, 410–414 (2013).

[b26] Hirori H., Doi A., Blanchard F. & Tanaka K. Single-cycle terahertz pulses with amplitudes exceeding 1 MV/cm generated by optical rectification in LiNbO_3_. Appl. Phys. Lett. 98, 091106 (2011).

[b27] Blanchard F. . Generation of 1.5 μJ single-cycle terahertz pulses by optical rectification from a large aperture ZnTe crystal. Opt. Express 15, 13212–13220 (2007). 19550589 10.1364/oe.15.013212

[b28] Yeh K. L., Hoffmann M. C., Hebling J. & Nelson K. A. Generation of 10 μj ultrashort terahertz pulses by optical rectification. Appl. Phys. Lett. 90, 171121 (2007).

[b29] Ropagnol X., Blanchard F., Ozaki T. & Reid M. Intense terahertz generation at low frequencies using an interdigitated ZnSe large aperture photoconductive antenna. Appl. Phys. Lett. 103, 161108 (2013).

[b30] Sell A., Leitenstorfer A. & Huber R. Phase-locked generation and field-resolved detection of widely tunable terahertz pulses with amplitudes exceeding 100 MV/cm. Opt. Lett. 33, 2767–2769 (2008). 19037420 10.1364/ol.33.002767

[b31] Jepsen P. U. & Fischer B. M. Dynamic range in terahertz time-domain transmission and reflection spectroscopy. Opt. Lett. 30, 29–31 (2005). 15648627 10.1364/ol.30.000029

[b32] Wu Q. & Zhang X.-C. Free-space electro-optic sampling of terahertz beams. Appl. Phys. Lett. 67, 3523–3525 (1995).

[b33] Wu Q. & Zhang X.-C. Free-space electro-optics sampling of mid-infrared pulses. Appl. Phys. Lett. 71, 1285–1286 (1997).

[b34] Wu Q. & Zhang X.-C. 7 terahertz broadband GaP electro-optic sensor. Appl. Phys. Lett. 70, 1784–1786 (1997).

[b35] Sharma G., Singh K., Al-Naib I., Morandotti R. & Ozaki T. Terahertz detection using spectral domain interferometry. Opt. Lett. 37, 4338–4340 (2012). 23073455 10.1364/OL.37.004338

[b36] Lu X. & Zhang X.-C. Balanced terahertz wave air-biased-coherent-detection. Appl. Phys. Lett. 98, 151111 (2011).

[b37] Dai J., Xie X. & Zhang X. C. Detection of broadband terahertz waves with a laser-induced plasma in gases. Phys. Rev. Lett. 97, 103903 (2006). 17025819 10.1103/PhysRevLett.97.103903

[b38] Sharma G. . Self-referenced spectral domain interferometry for improved signal-to-noise measurement of terahertz radiation. Opt. Lett. 38, 2705–2707 (2013). 23903117 10.1364/OL.38.002705

[b39] Cao X. D. & Meyerhofer D. D. Frequency-domain interferometer for measurement of the polarization mode dispersion in single-mode optical fibers. Opt. Lett. 19, 1837–1839 (1994). 19855670 10.1364/ol.19.001837

[b40] Singh K., Dion C., Lesk M. R., Ozaki T. & Costantino S. Spectral-domain phase microscopy with improved sensitivity using two-dimensional detector arrays. Rev. Sci. Instrum. 82, 023706 (2011). 21361600 10.1063/1.3556787

[b41] Hebling J., Stepanov A. G., Almási G., Bartal B. & Kuhl J. Tunable THz pulse generation by optical rectification of ultrashort laser pulses with tilted pulse fronts. Appl. Phys. B 78, 593–599 (2004).

[b42] Fülöp J. A. . Generation of sub-mJ terahertz pulses by optical rectification. Opt. Lett. 37, 557–559 (2012). 22344105 10.1364/OL.37.000557

[b43] Sato K. & Adachi S. Optical properties of ZnTe. J. Appl. Phys. 73, 926–931 (1993).

[b44] Wu Q. & Zhang X.-C. Ultrafast electro-optic field sensors. Appl. Phys. Lett. 68, 1604–1606 (1996).

[b45] Schall M., Walther M. & Jepsen P. U. Fundamental and second-order phonon processes in CdTe and ZnTe. Phys. Rev. B 64, 094301 (2001).

