# Supplementary material for: Ultra-high dynamic range electro-optic sampling for detecting millimeter and sub-millimeter radiation
Source: Sci Rep. 2016 Mar 15;6:23107. doi: 10.1038/srep23107 (PMC4791559; doi:10.1038/srep23107)
Supplement: Supplementary Information [file srep23107-s1.pdf]

Ultra-high dynamic range electro-optic sampling for detecting millimeter and sub-millimeter radiation

Akram Ibrahim[1,*], Denis Férachou[1], Gargi Sharma[2], Kanwarpal Singh[3], Marie Kirouac-Turmel[1]&Tsuneyuki Ozaki[1,*]

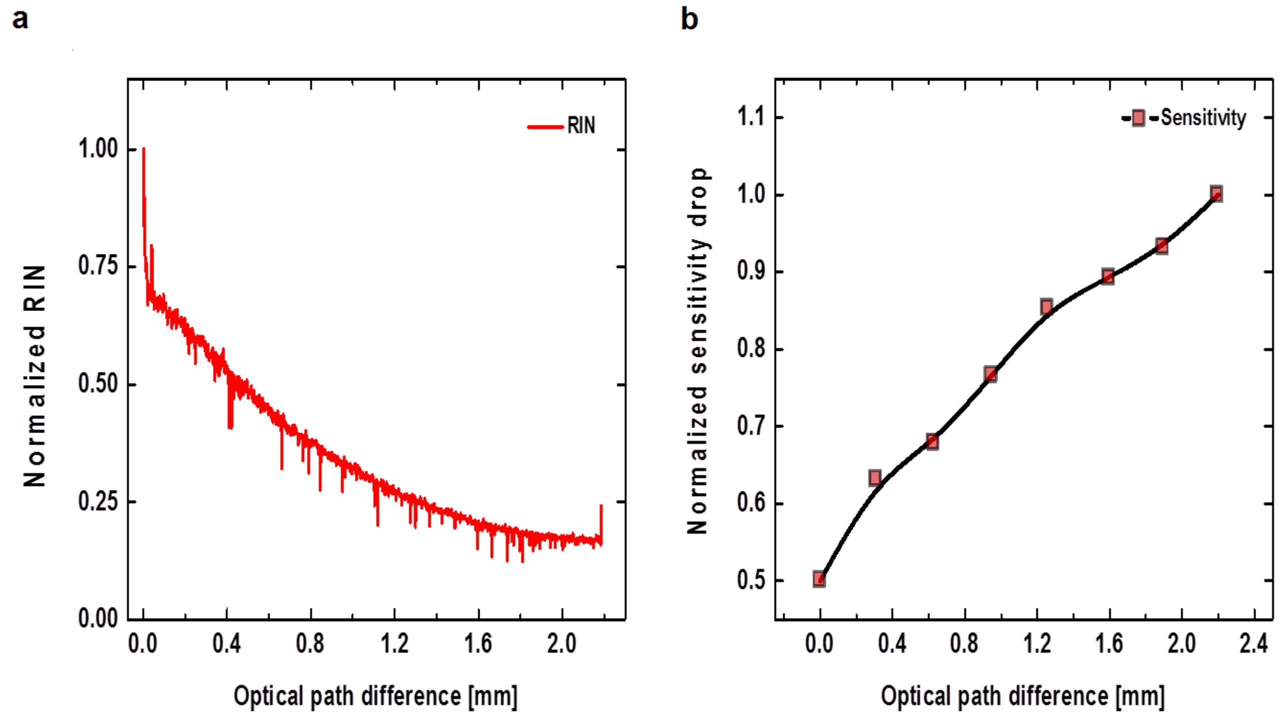

Supplementary Figure S1| Dependence of noise and sensitivity of spectral-domain interferometry technique on optical path difference between the interfering signals. (a) Normalized RIN and **(b)** normalized sensitivity drop are plotted against optical path difference (OPD). The RIN is high for smaller OPD whereas the drop in the sensitivity of the detection technique is low for smaller OPD.

Supplementary Note 1 Spectral-domain interferometry technique sensitivity and noise variations with optical path difference between the interfering signals. Error in phase measurement is dominated by relative intensity noise (RIN) at lower OPD whereas at higher OPD it is dominated by low sensitivity. RIN is calculated as standard deviation of the Fourier transformed data measured at the spectrometer over 60 seconds. RIN primarily arises due to intensity fluctuations in the laser pulses and is different for different laser sources. Sensitivity drop is a fundamental problem in spectral-domain interferometery and arises due to limited bandwidth falling on each pixel of the camera. Limited size of the camera pixels, causes averaging of the interferometric fringes leading to lower fringe contrast or higher sensitivity drop at longer OPD where modulation frequency is higher. A balance is achieved somewhere between minimum OPD and maximum OPD where phase measurement error is minimum. Supplementary Figure S1 shows that the RIN is higher for smaller OPD whereas the sensitivity of the SDI detection technique is enhanced for smaller OPD.
